# Healing from the Heart: A Burnout Prevention and Resilience Curriculum Innovation for Health Professionals in Underserved Areas

**DOI:** 10.3390/healthcare14172735

**Published:** 2026-08-27

**Authors:** Catherine Justice, Jordan McWilliams, Roger Brown, Virginia Fowkes, Joshua D. Kamimoto, Iris Price, Ivan Gomez, Sara Poplau, Mark Linzer

**Affiliations:** 1 Hennepin Healthcare Systems, Minneapolis, MN 55415, USA; mark.linzer@hcmed.org; 2 Hennepin Healthcare Research Institute, Minneapolis, MN 55415, USA; jordan.mcwill@gmail.com (J.M.); sara.poplau@hcmed.org (S.P.); 3 School of Nursing, Medicine, and Public Health, University of Wisconsin, Madison, Madison, WI 53705, USA; medicalresearchconsult@yahoo.com; 4 California Statewide AHEC Program, Fresno, CA 93701, USA; vfowkes@stanford.edu (V.F.); joshua.kamimoto@ucsf.edu (J.D.K.); price.iris@gmail.com (I.P.); 5 San Francisco Fresno Medical Education Program, University of California, Fresno, CA 93701, USA; ivan.gomez@ucsf.edu; 6 School of Medicine, Stanford University, Stanford, CA 94305, USA

**Keywords:** burnout, healthcare worker, resilience, system change, underserved, whole-person health

## Abstract

**Highlights:**

**What are the main findings?**

- Development of a whole-person approach to resilience for health professionals, trainees, and other healthcare workers in medically underserved areas.
- Measures supported the feasibility, acceptability, and utility of the curriculum, with appreciation for mindfulness, mind/body skills, community support, and system change aspects.

**What are the implications of the main findings?**

- The customizable, multi-level (individual, community, and system) curriculum and whole-person focus may have contributed to the successful piloting of the intervention.
- More system-focused interventions are likely needed to impact burnout scores.

**Abstract:**

**Background/Objectives**: Healthcare burnout is a pervasive problem affecting both professionals and patients. Resilience training may be especially important for healthcare teams practicing in underserved areas. This article describes the development and pilot testing of a curriculum innovation developed as part of California Area Health Education Centers’ (CA AHEC) resilience training program. **Methods**: A team of experts from CA AHEC and Hennepin Healthcare Systems led the development of a whole-person approach to resilience for health professionals, trainees, and other healthcare workers in medically underserved areas. A curriculum was piloted with 7 two-hour Train-the-Trainer video conferences and 1 six-hour in-person workshop. Participants (*n* = 44) included representatives from 12 CA AHEC centers. Attendance was tracked, and pre/post Knowledge, Skills, and Attitudes (KSA) and Mini Z Burnout Reduction surveys were administered. An engagement survey was fielded after training. **Results**: Attendance averaged 26 participants per session. Engagement surveys reflected high engagement, utility, and program appreciation, with KSA surveys reflecting large gains in understanding burnout mechanisms/prevention strategies (13.5% high pre- vs. 94.7% post-, *p* < 0.001; Absolute difference of 81.22, 95% CI 66.87–95.58), large Cohen’s *h* Effect Size (ES) of 1.9) and the perceived ability to use the training to cope with stress (8.11% high pre- vs. 94.7% post, *p* < 0.001; Absolute Difference of 86.63%, 95% CI 73.55–99.71, large Cohen’s *h* ES of 2.1). Mini-Z burnout scores remained relatively unchanged. Mindfulness, mind/body approaches to resilience, community support, and system change were rated as the most impactful curricular aspects. **Conclusions**: This whole-person resilience curriculum represents an emerging approach to healthcare burnout. While the curriculum was greatly appreciated by attendees, working conditions and burnout remained unchanged, suggesting a need for programs that address underlying systemic stressors.

## 1. Introduction

Burnout is a long-term stress reaction creating emotional exhaustion, depersonalization, and a declining sense of personal accomplishment [1,2]. It is prevalent nationwide across all areas of healthcare with detrimental consequences on professionals and the overall system [3]. This includes associations with poorer quality of care, unsustainable healthcare worker (HCW) roles, high turnover rates, and reduced patient access to healthcare [4]. Particularly at risk are HCWs working in underserved communities with limited resources [5,6]. Few health systems treating underserved populations have implemented comprehensive approaches to combat burnout that address individual, community, and system-level factors [6]. Researchers have tested various strategies to reduce burnout in other settings, including mindfulness, self-care, lifestyle, and resilience programs, with a positive impact on burnout, stress, mental health, absenteeism, and quality of life [7,8,9,10,11]. While results from these myriad approaches are generally positive, there is limited reporting on interventions that use a multi-level approach to burnout, with individual-, community-, and system-level interventions all represented [12]. Additionally, most of the reported programs use a set curriculum and delivery model, with little to no ability to customize the intervention to individual needs.

The California Statewide Area Health Education (CA AHEC) trains all healthcare workforce roles, including students, residents, practicing health professionals, and other patient-facing professionals, to improve access and quality in medically disadvantaged communities. CA AHEC is comprised of 12 centers located in underserved communities throughout the state. Collectively, they partner with over 700 educational, clinical, and related community-based and government organizations to conduct their programs [13]. Nine centers are hosted by community health center consortia, two by family medicine residency programs, and one at a non-profit corporation. These centers face challenges including serving low-income/low-resourced populations, low reimbursement rates, clinically complex patient populations, and staffing shortages.

Responding to healthcare burnout during the COVID-19 pandemic, CA AHEC and statewide leaders developed a program for burnout prevention and resilience training. AHEC leadership engaged national experts at Hennepin Healthcare’s Institute for Professional Worklife (IPW) to collaborate on the development of a comprehensive program responsive to the needs of its 12 community-based sites (AHEC centers) throughout the state. The objective of this study was to conduct a program evaluation of the development and pilot testing of a curriculum innovation, developed as part of CA AHEC’s program, and a Train-the-Trainer (TtT) pilot with participants from multiple AHEC centers.

## 2. Materials and Methods

### 2.1. Curriculum Description

**Figure 1:**
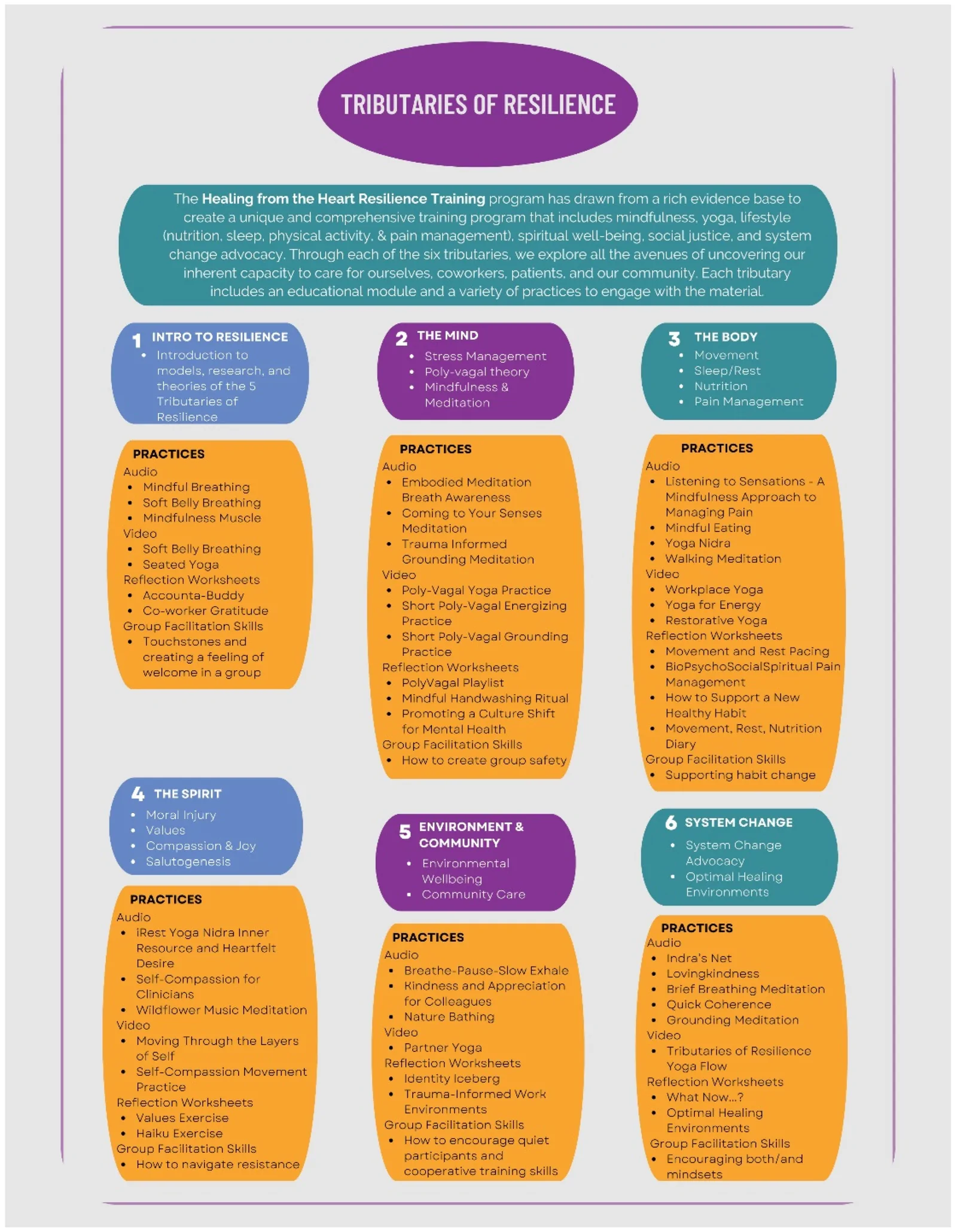
Healing from the Heart: Resilience Training for Healthcare Workers curriculum, practices, and Train-the-Trainer facilitation techniques.

Healing from the Heart: Resilience Training for Healthcare Workers curriculum was developed with psychological, physical, spiritual, community, environmental, and system frameworks in mind, bringing a multimodal, whole-person approach to address the complex nature of burnout. A key innovation to the program was acknowledging system drivers of burnout alongside personal stress management practices. Integrative Health and health equity principles were applied within the curriculum, including evidence-based Traditional Complementary and Integrative Medicine (TCIM) modalities (mindfulness, meditation, and yoga), lifestyle (nutrition, sleep, physical activity), and stress management practices with a trauma-informed, multi-level focus (individual, community, and system) [14]. TCIM modalities were chosen based on their use in other successful resilience programs [10,15,16,17,18,19] and the expertise of the curriculum developers/facilitators. A series of six modules was created, each with a menu of practice options (Figure 1). Modules included: Introduction to Resilience, Mind, Body, Spirit, Community and Environment, and System Change. To address a wide diversity of HCW needs and preferences, a menu of practice options was created, including guided meditations (21), movement practices such as chair yoga, mindful walking, and restorative yoga (12), and reflection worksheets such as a mindful hand-washing prompt, reflections on workplace culture, lifestyle change support, values identification, and creative reflection practices (15). Recognizing the time pressures faced by HCWs, curriculum practices were structured with variable durations to facilitate asynchronous use by individuals or groups based on their specific needs. For example, the curriculum embraced both didactic and experiential components and included short practices that could be conducted during busy workdays. The TtT program included the Healing from the Heart curriculum, alongside synchronous training and additional curriculum on group facilitation techniques (how to create group welcome and safety, motivational interviewing basics, trauma-informed group dynamics, etc.) to prepare the trainees to effectively lead groups and share the curriculum with their centers (Figure 1).

### 2.2. Train-the-Trainer Program

The resilience curriculum was pilot-tested with TtT cohorts of 44 individuals, recruited as a convenience sample from 12 centers, spanning 27 distinct job roles (directors, managers, coordinators, and patient care staff representing workforce development, behavioral/mental health, equity, quality assurance, programming, and research). Seven sessions were conducted online, followed by an in-person workshop hosted at AHEC centers throughout California. The TtT cohorts met virtually during workdays for seven 2-h sessions, culminating in a single 6-h in-person workshop. Cohorts 1 and 2 took place separately between September 2023 and January 2024 and between September 2024 and January 2025. Trainings were facilitated by a Doctor of Physical Therapy (author CJ) with support from a project coordinator (author JM). For the second cohort, a third facilitator included one of the trainees from the original TtT cohort. Each session included reflection on the curriculum, group connection practices, facilitation training, and discussion (Figure 1). The first six sessions followed the curriculum from the pre-recorded modules, and the final online session and in-person workshop focused on its application within work environments and the development of action plans for the distribution of the curriculum throughout AHEC centers. At the request of the trainees, a series of hour-long post-training check-in virtual meetings occurred between March 2024 and July 2025 to allow trainees to connect, ask questions, and share how they were utilizing the curriculum. Project ECHO’s “All Teach, All Learn” model [20] was utilized throughout, establishing a culture of humility, engagement, respect, and mutual learning among participants and facilitators. This program was granted exempt status by Institutional Review Boards at the University of California, San Francisco, and Hennepin Healthcare Research Institute.

### 2.3. Outcome Measures

The Mini-Z Worklife and Burnout Reduction Instrument [21] was used to measure outcomes and drivers of burnout within the cohorts before (Pre-) and after the virtual training (Post-1) (Supplementary Material S1). The Mini-Z is a validated instrument consisting of 10 items rated on a 0–5 Likert scale and one open-ended question. Additional questions were asked if the participant indicated they were either a student, resident, or Fellow (Mini ReZ) OR in Administration (Ad-Mini), based on how they responded to the demographic questions “Which of the following best describes you (choose one)? and/or the question “Do you have an administrative leadership role?”. A Knowledge, Skills, and Attitudes (KSA) questionnaire and an engagement survey were created specifically for this program. The use of unvalidated measures was deemed acceptable for the program evaluation study design to tailor the outcome measure to the curriculum. The KSA was administered before (Pre-), after the virtual training (Post-1), and one month later, after the in-person workshop (Post-2), surveying participants on their perceptions of their ability to recognize and manage stress and burnout (Supplementary Material S2). An engagement questionnaire was also administered after the virtual training (Post-1) and after the in-person workshop (Post-2), asking participants which modules they accessed, the most useful parts of the training, and open-ended questions soliciting ideas on how to improve the program (Supplementary Material S3). For participants who completed multiple versions of a survey, the version with the most complete data was included for analysis.

In addition to these structured outcome measures, a tracking document was created to record how participants utilized the curriculum after the training. This document was updated from post-training virtual check-in conversations, email solicitations, and informal interviews with participants.

### 2.4. Analysis

Outcomes from the two cohorts were combined for all analyses, except for the engagement survey, for which responses from each cohort were analyzed separately in addition to a combined score. For participants who completed multiple versions of a survey, the submission with the most answered questions was used in analysis. Many of the duplicates were largely blank and discarded. If duplicates had a similar level of completion, the earliest survey submitted was used for pre- and the latest for post- in the analysis.

Pre–post changes in binary outcomes were analyzed using a partially paired Wald test, which accommodates mixtures of complete pre–post pairs and incomplete observations. Standard methods were not suitable because McNemar’s test discards incomplete cases, while independent proportion tests ignore within-subject correlation. Using the Derrick et al. [22,23] framework, paired and unpaired observations contributed to a single variance estimator for the marginal proportional difference between post- and pre-training assessments. The method adjusts for correlation among complete pairs and naturally spans the range from fully paired to fully independent data. Wald statistics and 95% confidence intervals were computed in Stata version 19 using a custom implementation for binary outcomes.

Open-ended survey responses were analyzed using ChatGPT-5.5 Instant UCSF Enterprise, an AI-powered large language model. The system was prompted to conduct a qualitative analysis using inductive thematic analysis (transcription, selection of keywords, coding, theme development, conceptualization, and development of a conceptual model) [24,25]. Responses were reviewed iteratively by a single investigator and coded at the semantic level to identify recurring concepts related to participants’ experiences with the TtT program. AI-generated themes were reviewed and verified against the raw data by a single investigator (author JK). Themes were subsequently verified by asking the AI model to repeat what data were used and to re-verify it against the raw data.

## 3. Results

**Figure 2:**
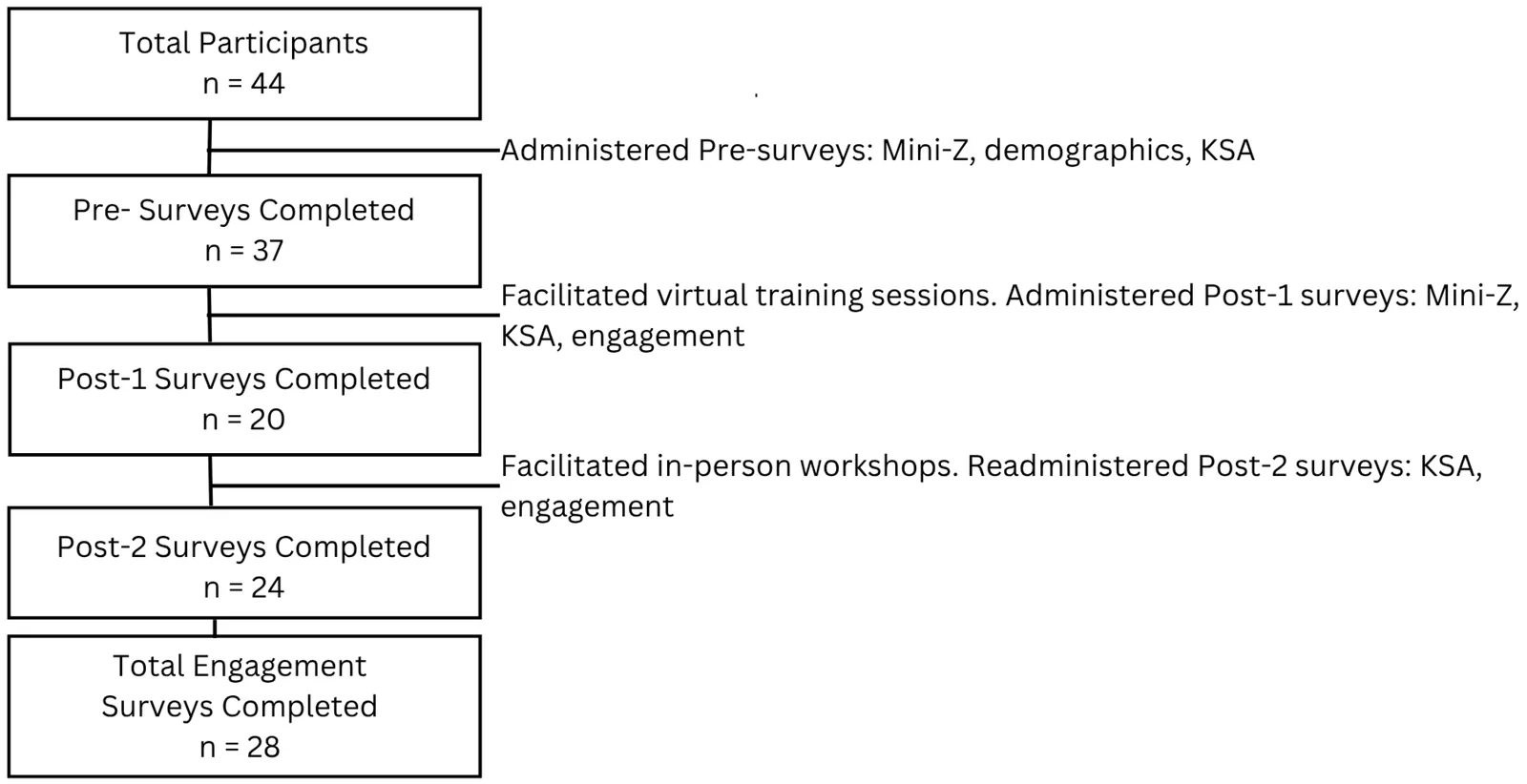
Healing from the Heart program flowchart. Note: KSA = Knowledge, Skills, and Attitudes Survey; Mini-Z = Mini-Z Worklife and Burnout Reduction Instrument.

Of 44 enrolled participants, 37 completed demographics and pre-surveys, 20 completed Post-1 surveys, and 24 completed Post-2 assessments (Figure 2).

Demographics surveys revealed 31 participants working full-time jobs, with 32.4% working hybrid in-person and remotely (Table 1). Respondents identified as women (70.3%), and Hispanic/Latino/Latinx (48.6%), with 45.9% identifying as White. Participants were a diverse group of health care workers, with 27 distinct job roles spanning leadership, administrative, community, and clinical domains. Six participants were health justice equity fellows, a program for students in their gap year between undergraduate and graduate work. These participants came from the AHEC serving one of the most impoverished inner-city areas. Attendance averaged 26 participants per virtual session (ranging 19–36) and 5 participants at each in-person workshop (ranging 2–7) (Table 2). The overall median number of virtual sessions attended by each participant was four (IQR 2–6). Separately, Cohort 1 had a median attendance of four participants (IQR 2–5), and Cohort 2 had a median attendance of five participants (IQR 3–6). Four participants joined the in-person workshops virtually.

Feedback from post-engagement surveys at both time points (Post-1/Post-2) was combined to illustrate the full engagement of the program (*n* = 28, Table 3). Results indicated that the majority of participants watched all six modules and selected all modules as relevant to their work and personal development. The first cohort rated Module 5 (Community and Environment) and Module 6 (System Change) as most impactful, whereas the second cohort rated Module 2 (The Mind) and Module 3 (The Body) as most impactful. For participants who missed live TtT sessions, 35% reported watching all missed session recordings, 45% watched some, and 20% did not watch any of the recordings. Over half the respondents reported utilizing movement videos and reflection worksheets, and about one-third reported listening to the audio practices. Over three-quarters of respondents found mindfulness practices very or extremely effective for stress reduction. Almost all participants were at least Somewhat Satisfied with the overall program, with over half Extremely Satisfied.

A total of 31 open-ended survey responses were analyzed across two cohorts (*n* = 31) of the program. Six major qualitative themes were identified: mindfulness and meditation, practical application of skills, resilience and burnout prevention, reflection and self-awareness, scheduling and accessibility, and knowledge sharing. Word-frequency analysis demonstrated that terms related to time, work, mindfulness, attendance, and perceived helpfulness occurred most frequently. Participants mentioned concepts of mindfulness and meditation most often in conjunction with centering practices and breathwork. For practical application, respondents reported immediately applying techniques provided in both their daily work and personal life. The techniques most applied were mindful pauses, breathing exercises, movement breaks, and self-care practices. As it pertains to resilience and burnout prevention, participants reported an improved understanding of the mechanisms that lead to burnout and of strategies that build greater resilience, providing more effective coping tools and language for discussing burnout. Feedback on reflection and self-awareness produced comments that emphasized intentional reflection, awareness of personal needs, work–life balance, and mind–body connection. Consensus on scheduling and accessibility yielded consistent results, with most participants noting that the time was appropriate. Participants also reported common barriers to attendance, including meetings, deadlines, conferences, family obligations, and the length of Zoom sessions. Within the theme of knowledge sharing, participants reported sharing resources and practical skills with colleagues, fellow team members, and family members.

**Figure 3:**
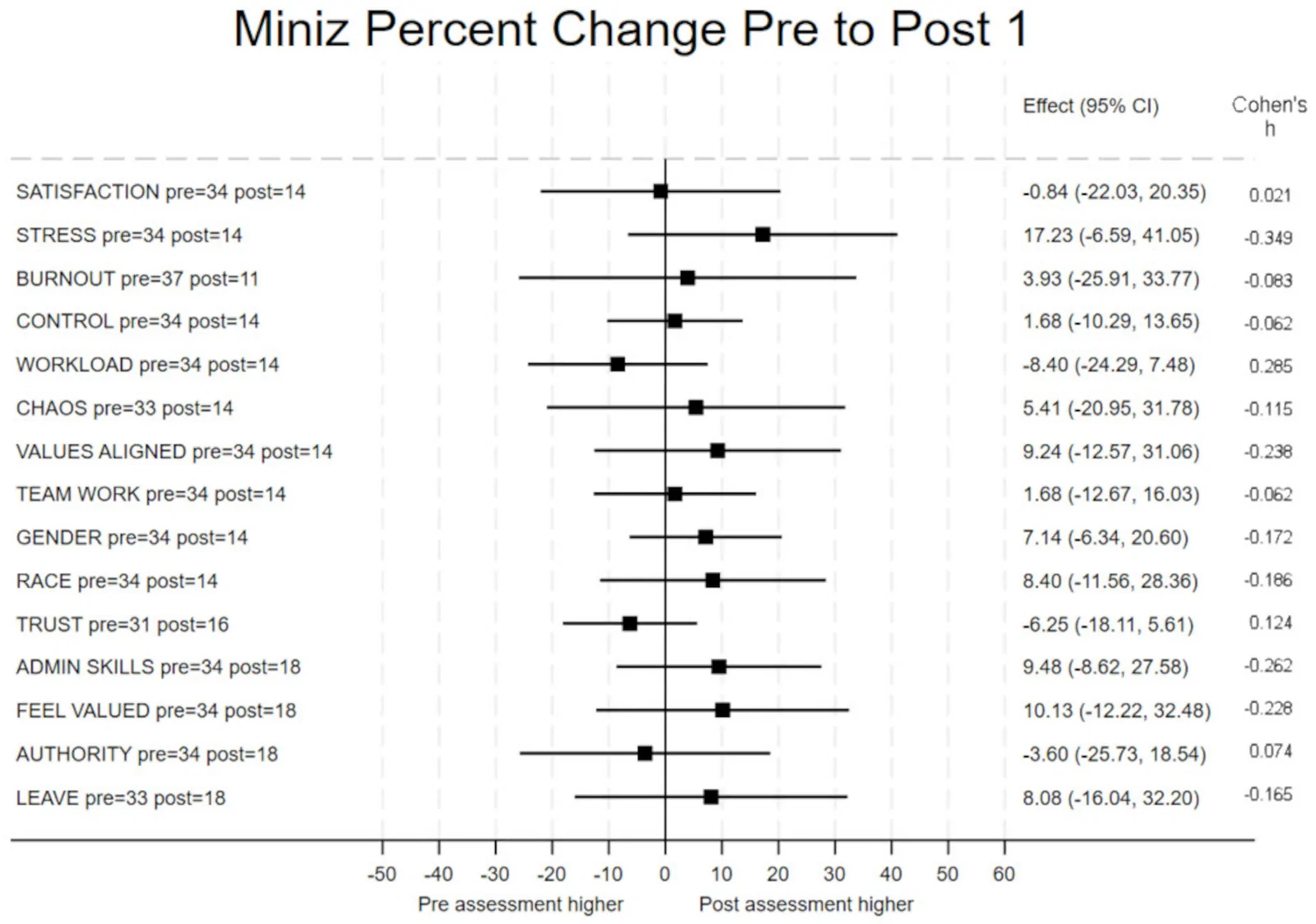
Forest plot of Mini-Z change after training. Note: The line represents the 95% Confidence Intervals (CI), and the square in the center of the line represents the percent difference.

Pre–post training changes in Mini-Z outcomes were examined using partially paired Wald tests to account for a combination of repeated and unmatched respondents (Pre *n* = 37, Post-1 *n* = 18) (Figure 3). Overall, Mini-Z domains remained relatively stable over time, with no statistically significant changes observed, although wide confidence intervals indicate limited precision and potential lack of power to detect modest changes. Perceived stress increased from 47.1% high stress to 64.3%, representing the largest unfavorable change, although this increase did not reach statistical significance. Several domains showed favorable but nonsignificant trends, including professional values alignment, workplace skills, and feeling valued, a known burnout mitigator [26].

**Figure 4:**
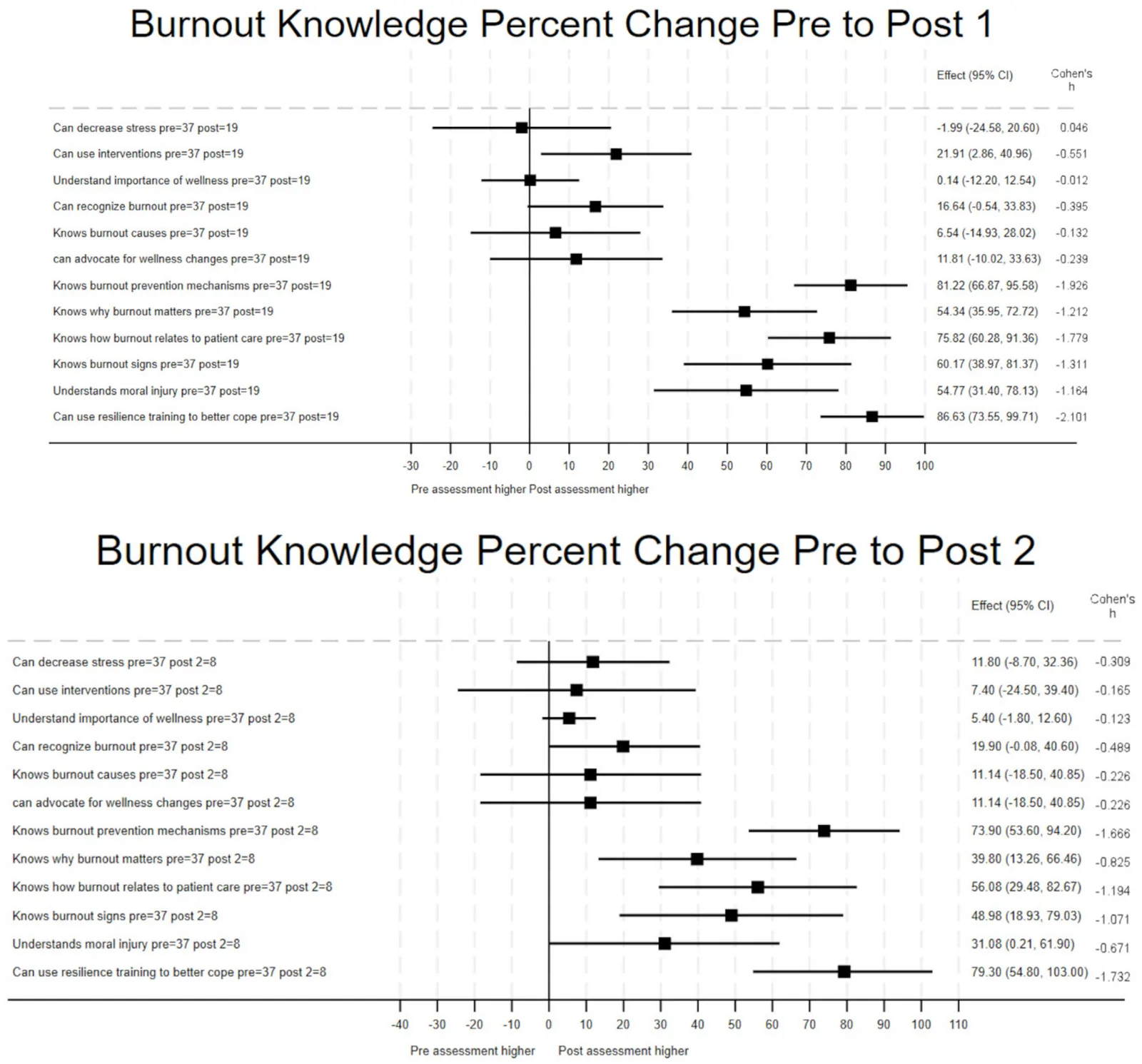
Forest plot of burnout knowledge and attitudes change after training. Note: The line represents the 95% Confidence Intervals (CI), and the square in the center of the line represents the percent difference.

**Figure 5:**
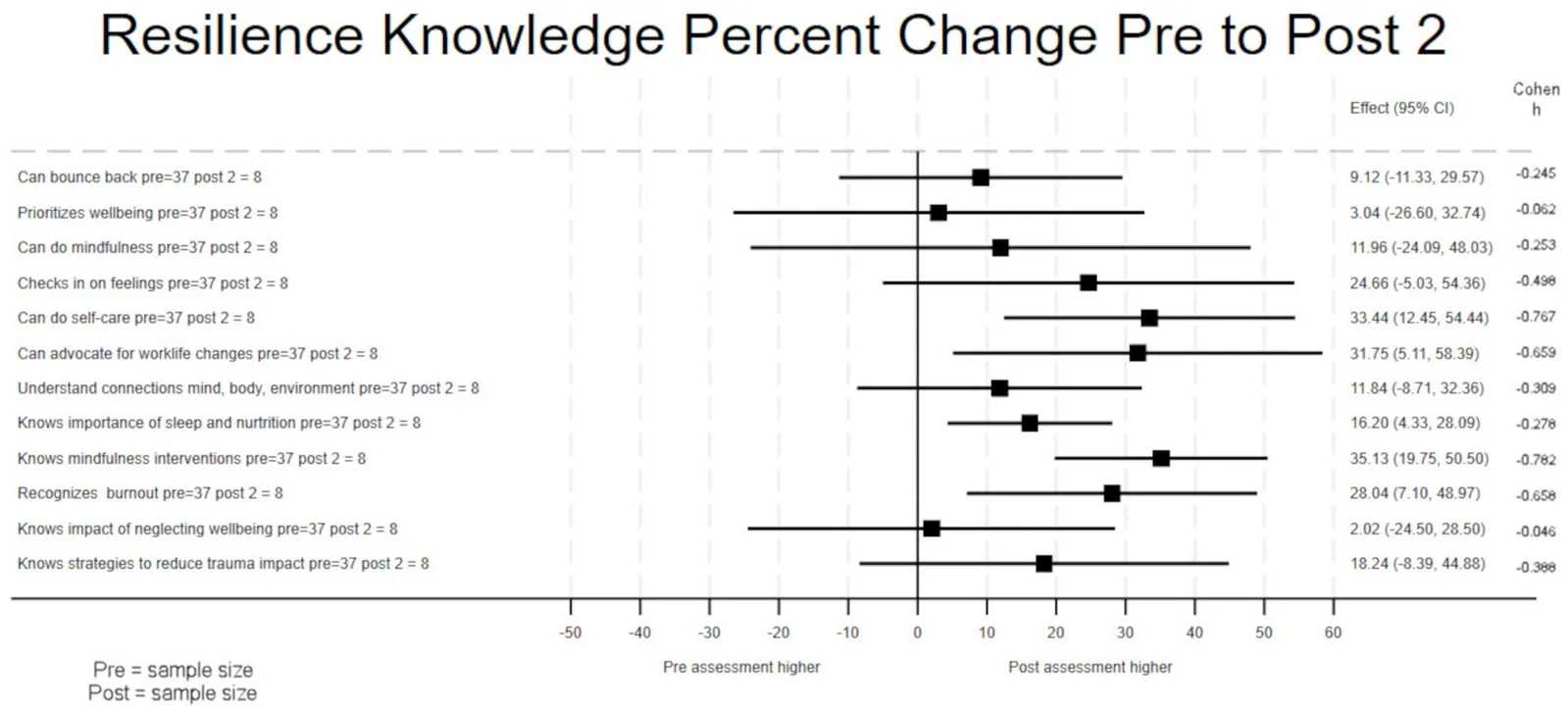
Forest plot of resilience knowledge and attitudes change after training. Note: The line represents the 95% Confidence Intervals (CI), and the square in the center of the line represents the percent difference.

KSA surveys demonstrated substantial gains in perceptions of educational and conceptual knowledge domains (Pre *n* = 37, Post-1 *n* = 19, Post-2 *n* = 8) (Figure 4 and Figure 5). For burnout knowledge, the largest effect sizes were observed in perceptions of how to use the training to cope with stressors, understanding burnout mechanisms and prevention strategies, understanding the importance of burnout prevention, recognizing relationships between burnout prevention and patient outcomes, recognizing signs and symptoms of burnout, and understanding moral injury (Figure 4).

Smaller, non-significant changes were observed for the perceived ability to reduce workplace stressors, empowerment to address workplace burnout issues, and confidence advocating for evidence-based workplace changes. Perceptions of recognition of burnout symptoms in oneself and others showed a positive trend approaching statistical significance (*p* = 0.058), while understanding the importance of personal wellbeing was high at both time points.

Self-reported resilience knowledge scores improved across multiple domains following the training (Figure 5). The largest effect sizes were observed in perceived confidence in implementing self-care strategies in times of stress, understanding resilience and mindfulness concepts, self-compassion during stressful situations, confidence engaging in mindfulness practices, recognizing burnout symptoms, and advocating for workplace wellbeing changes. Several knowledge items that may have been limited by ceiling effects at post-test included understanding connections among mind, body, and environment and recognizing the importance of sleep, nutrition, movement, and stress management, with 100% endorsement after the training.

Action plans created by participants during the in-person workshops and monthly check-ins included intentions to build personal wellness goals around movement and stress management, create a “Resiliency Corner” in employee communications, include the curriculum in employee wellness initiatives, participate as a co-facilitator for future TtT cohorts, implement the curriculum within their centers, incorporate a pathway program for students, provide burnout and relief spaces for staff, build safer communities and support for practices, implement learned strategies and resources into staff toolkits, and develop personal wellness goals for sleep, stress reduction, and movement. Feedback from interviews with three AHEC center directors who participated as trainees in the first cohort indicated the usefulness of the training personally and professionally and the applicability of the curriculum for their centers. They reported experiencing a supportive environment during training and an appreciation for the menu of options and curriculum adaptability. They suggested gathering leadership support for the training and developing applications with internal teams (e.g., consortia newsletter topics). They recommended training additional cohorts such as leaders from AHEC partnership sites and AmeriCorps students, developing a recruitment flyer and podcasts, and defining system change needs within their centers.

## 4. Discussion

The Healing from the Heart Train-the-Trainer program was feasible to implement and well attended, with high use of the guided movement videos and reflection practices, and appreciation for the mindfulness practices and curriculum. KSAs shifted considerably, with increased perceptions of the ability to practice mindfulness, emotional awareness, reduce the impact of trauma, understand the drivers of burnout and moral injury, and improve coping strategies. Nonetheless, burnout remained stable throughout the training.

Attendance and post-engagement survey results support the feasibility of this program’s usage in a variety of settings. While outcomes showed consistent engagement, attendance declined as the program progressed. This type of attrition has been reported in similar programs [27]. Future programs may consider addressing the need for protected time in the workday to attend sessions and engage with the materials. The majority of participants who missed sessions reported watching the recordings, indicating the importance of offering busy HCWs multiple means of engagement. The qualitative analysis emphasizes the accessibility of the hybrid model utilizing a combination of live and recorded content. Use of both in-person and virtual formats is a similar approach to Rees et al.; however, their program was shorter in duration, with a four-hour in-person training followed by three virtual sessions [28]. The review by Scheuch et al. recommended that participants apply resilience skills in a variety of circumstances, something that a longer, more comprehensive program, as described here, may permit [12]. While most TtT participants accessed all modules, over one-quarter of participants accessed some but not all, underscoring the utility of an “à la carte” menu that may allow busy professionals and learners to focus on the most relevant topics for their needs. The qualitative data illustrated the facilitating aspects of the program as its flexibility (with a mix of real-time and pre-recorded materials) and the variety of practice options (movement, audio, and reflection worksheets). This diverse range of practices may have contributed to the curriculum’s broad appeal across various professions, levels of training, and experience. It is worth noting that while participants represented a wide range of healthcare professions, most were not practicing clinicians; only one was a physician who attended a single session. However, the informal interviews with AHEC center directors support the theory that those with administrative/leadership roles may be more likely to influence system changes.

This program builds on other healthcare resilience programs with similar approaches. The mindfulness and meditation practices were noted as particularly meaningful for participants, in line with research on Mindfulness-Based Stress Reduction programs [29]. Several other studies have incorporated mindfulness and TCIM practices into their programs with positive outcomes [8,9,10,28,30]. Others included a focus on community support [31], self-compassion [7], coping and stress management skills [32,33], and broader cognitive, emotional, physical, and spiritual strategies for wellbeing [34]. These methods are supported by the meta-analysis by Zhang et al., which demonstrates the effectiveness of incorporating yoga and other relaxation techniques into HCW well-being programs [35] However, few programs incorporate multi-level interventions as recommended in a scoping review of workplace resilience training programs by Scheuch et al. [12]. None, to our knowledge, has included a whole-person, multi-level approach to individual, community, and larger system-level resilience. This biological, psychological, social, and ecological systems approach is recommended in a recent review by Ungar et al. that describes the need for culturally relevant resources alongside individual practices [36]. Curriculum on trauma-informed care [37], equity in healthcare workspaces [38], optimal healing environments [39], the quintuple aim of healthcare [40], and addressing system drivers of burnout [41] were explored in the final two modules of the curriculum and TtT sessions.

Interestingly, the most popular modules for cohort 1 were those in Community and Environment and System Change, indicating that a community/system-level approach to burnout may be particularly appropriate for HCWs working with underserved populations. However, cohort 2 rated the Body and Mind modules as most impactful, indicating that personal practices supporting physical and mental/emotional health still may play an important role in supporting HCW’s working in under-resourced environments. While it is not possible to draw definitive conclusions about the differing preferences of the cohorts, multiple factors may have been influential. The addition of a third facilitator from a CA AHEC center for the second cohort may have shifted the trainees’ experience. Additionally, many external sources of stress rose in 2024 that may have impacted the needs of cohort 2. The observed shift in desired resources from system-level interventions towards body- and mind-focused support may reflect broader changes in the stress landscape. Recent national surveys suggest that political uncertainty, economic concerns, and societal issues remained prominent in 2024–2025 [42], while overall stress levels remained relatively stable [43,44,45,46]. These stressors may be perceived as less amenable to local organizational or system-level solutions, potentially increasing interest in resources that enhance individual coping, resilience, stress management, and well-being. However, because the survey did not directly assess the sources of the respondents’ stress and the lack of a control group, this interpretation remains speculative. In future studies, it will be worth exploring if participants prioritize self-care practices when system change may not feel possible.

KSA results for burnout and resilience demonstrated large effect sizes. These results provide preliminary support for the feasibility of the integrative, whole-person, multi-level nature of the curriculum. However, given the small sample size and attrition, these results should be interpreted with caution. Trends in improvements across self-reported Resilience and Burnout knowledge skills and attitudes were consistent from Post-1 to Post-2 time points, suggesting that the inclusion of the in-person workshop and/or the passage of time may have helped to consolidate gains. However, not all participants who completed the Post-2 survey attended the in-person workshop, which may account for the greater variation in results at the Post-2 time point. Additionally, the small Post-2 sample size does not allow for a definitive longitudinal assessment of program effectiveness.

Our results suggest that improvements in self-reported knowledge and confidence may not be associated with measurable reductions in burnout over this relatively short follow-up period. The lack of robust change in the Mini-Z data suggests that despite the perceived utility of the training, larger, system-level intervention may be needed to mitigate organizational drivers of burnout. But while self-reported burnout scores did not change, perceived stress did increase. We postulate that stable burnout in the presence of rising stress may indicate a potential protective effect of the curriculum, although our methodology does not allow for a direct inference. The training was intended to create a group of healthcare workers who better understand burnout prevention and resilience skills so that they could help others engage with the curriculum and advocate for individual, community, and system-change approaches within their organizations. Domains related to self-efficacy and workplace empowerment may require more intensive or sustained training approaches. Follow-up will be needed to learn how and where participants used the curriculum to assess the long-term impact of training on system factors contributing to burnout. An improved ability to understand the drivers of burnout, engage in self-care and community-focused well-being, and advocate for system change may lead to better working conditions and lower burnout scores over time. More robust follow-up studies are needed to confirm this hypothesis.

### Limitations and Future Directions

Several factors limit the generalizability of this program. As there was no control group, no cause-and-effect relationship could be ascertained between the intervention and the outcomes. Because the analysis was conducted for program evaluation purposes and the dataset was relatively small, findings should be interpreted as descriptive and exploratory rather than statistically generalizable. Selection bias could affect the outcomes, as participation in the program was voluntary. Only self-reported outcome measures were used, increasing the potential for social desirability bias, and the pre-/post-test design is vulnerable to response shift bias. The KSA and engagement surveys have not been independently validated, and no long-term data have been collected. Participants primarily held administrative and leadership positions, and front-line healthcare workers are under-represented. Attendance was recorded for the virtual sessions, but it is unknown how many participants watched session recordings and how that delivery model influenced outcomes. Engagement surveys were administered twice; however, not all participants attended the in-person workshop or completed the surveys at both time points. Differences in outcomes based on this have not been considered. Comparisons of group percentages were based on relatively small sample sizes; as such, percentage estimates can be unstable and highly sensitive to the outcomes of a few participants, resulting in substantial sampling variability and wide confidence intervals. Consequently, observed percentage differences between groups may overestimate or underestimate the true population differences and should be interpreted with caution. Replication in larger samples and other types of health professionals is needed to determine the robustness and generalizability of these findings.

Attrition is another factor to consider, as many participants did not complete the post- surveys. While dedicated time was given to complete the survey in the final session and in-person workshop, those who did not attend these sessions may have been less likely to complete the post-surveys. While surveys were emailed to all participants, no incentives were offered (such as a gift card or continuing education credits). Future studies should consider the use of survey incentives or dedicated follow-up to encourage more participation and/or compare characteristics of participants who completed surveys vs those who did not.

Because follow-up survey completion was incomplete, a sensitivity analysis was performed using Last Observation Carried Forward (LOCF) imputation. LOCF was selected because missing post-training assessments were primarily due to participant disengagement, making the assumption of no improvement a clinically and operationally plausible, and intentionally conservative scenario. Consistent with expectations, effect estimates from the LOCF analysis were generally smaller than those obtained from the primary partially paired analyses, which utilize all available paired and unpaired observations. Nevertheless, the direction and overall interpretation of the findings remained unchanged, supporting the robustness of the conclusions to plausible assumptions regarding missing outcome data.

Despite these limitations, the Healing from the Heart: Resilience Training for Healthcare Workers curriculum shows promise as a multi-level, integrative, whole-person approach to the complex and pervasive issue of HCW burnout. CA AHEC will continue promoting use of the curriculum, with ongoing distribution to its centers and other educational and clinical partners. Results from this piloted program will inform CA AHEC’s ongoing evaluation and utilization.

## 5. Conclusions

The Healing from the Heart training was found to be feasible and impactful, with surveys demonstrating high engagement and appreciation. In particular, data from the KSA survey indicate improved perceptions of understanding of and ability to prevent burnout and manage workplace stressors. The whole-person, integrative, multi-level (individual, community, and system) approach is a unique feature of the curriculum that may have contributed to the positive results. Coupling individual/community resilience trainings with system-focused programs to alter work environments may ultimately be the most effective approach. Longer studies with control groups and larger sample sizes are needed to confirm these results. While ongoing evaluation of its use and impact continues, current experience suggests that multi-level, whole-person approaches such as the Healing from the Heart curriculum may help address the complex problem of HCW burnout in underserved communities.

## Figures and Tables

**Table 1:** Demographic characteristics of 37 participants in Healing from the Heart Resilience Train-the-Trainer program.

Variable (*n* = 37)	*n* (%)
Work Setting	
In-person	14 (37.8%)
Remote	5 (13.5%)
Hybrid	12 (32.4%)
Gender Identity	
Male	5 (13.5%)
Female	26 (70.3%)
Non-binary	3 (8.1%)
Other	1 (2.7%)
Hispanic/Latino/Latinx	
Yes	18 (48.6%)
No	17 (45.9%)
Other	1 (2.7%)
Race	
Asian	7 (18.9%)
Black or African American	2 (5.4%)
Middle Eastern or North African	2 (5.4%)
Native American or Alaska Native	3 (8.1%)
Native Hawaiian or Other Pacific Islander	1 (2.7%)
White	17 (45.9%)
Other	2 (5.4%)
Prefer not to answer	3 (8.11%)
Urban/Rural	
Urban	29 (78.4%)
Rural	1 (2.7%)
Don’t know	4 (10.8%)
Age Range	
20–29	5 (13.5%)
30–39	17 (45.9%)
40–49	6 (16.2%)
50–59	3 (8.1%)
60–69	3 (8.1%)
Organizational Role	
Administrative staff	31 (83.8%)
Clinical staff	3 (8.1%)
Fellow	2 (5.4%)
Health professions student	1 (2.7%)
Highest Degree	
Doctorate or professional degree	3 (8.1%)
Master’s degree	15 (40.5%)
Bachelor’s degree	12 (32.4%)
Associate’s degree	2 (5.4%)
Certificate Program	2 (5.4%)
Some college, no degree	3 (8.1%)

Some participants skipped or left certain variables blank, resulting in fewer responses and total percentage scores of less than 100%.

**Table 2:** Attendance of 44 participants by session in Healing from the Heart Resilience Train-the-Trainer program cohorts combined.

Session Title	Attendance, *n* (%)
Module 1: Intro to Resilience ^a^	36 (81.8%)
Module 2: The Mind ^a^	26 (59.1%)
Module 3: The Body ^a^	31 (70.5%)
Module 4: The Spirit ^a^	26 (59.1%)
Module 5: Environment and Community ^a^	24 (54.5%)
Module 6: System Change ^a^	22 (50%)
Module 7: Putting it All Together ^a^	19 (43.2%)
In-person workshops/retreats (4 combined)	19 (43.2%)

^a^ Sessions delivered virtually.

**Table 3:** Engagement survey results of cohort 1 participants (*n* = 12) and cohort 2 participants (*n* = 16) in the Healing from the Heart Resilience Train-the-Trainer program.

	Participant Response, *n* (%)						
Survey Item		Module 1	Module 2	Module 3	Module 4	Module 5	Module 6
Did you interact with any of the module files using the link that was sent to you? If so, select the modules you accessed through the links.	Cohort 1	12 (100%)	11 (91.7%)	12 (100%)	11 (91.7%)	12 (100%)	10 (83.3%)
	Cohort 2	16 (100%)	13 (81.3%)	16 (100%)	12 (75%)	11 (68.8%)	10 (62.5%)
	Combined	28 (100%)	24 (85.7%)	28 (100%)	23 (82.1%)	23 (82.1%)	20 (71.4%)
		Module 1	Module 2	Module 3	Module 4	Module 5	Module 6
Which sessions did you find relevant to your work or personal development? Select all that apply.	Cohort 1	10 (83.3%)	10 (83.3%)	10 (83.3%)	9 (75%)	11 (91.7%)	10 (83.3%)
	Cohort 2	12 (75%)	16 (100%)	14 (87.5%)	14 (87.5%)	13 (81.3%)	11 (68.8%)
	Combined	22 (78.6%)	26 (92.9%)	24 (85.7%)	23 (82.1%)	24 (85.7%)	21 (75%)
		Module 1	Module 2	Module 3	Module 4	Module 5	Module 6
Were there modules that you found most exciting or impactful? Select all that apply.	Cohort 1	6 (50%)	8 (66.7%)	8 (66.7%)	7 (58.3%)	9 (75%)	9 (75%)
	Cohort 2	5 (33.3%)	11 (73.3%)	10 (66.7%)	8 (53.3%)	7 (46.7%)	6 (40%)
	Combined	11 (40.7%)	19 (70.4%)	18 (66.7%)	15 (55.6%)	16 (59.3%)	15 (55.6%)
		All	Some	None			
Did you watch any of the recordings of the live sessions you missed?	Cohort 1	4 (44.4%)	2 (22.2%)	3 (33.3%)			
	Cohort 2	3 (27.3%)	7 (63.6%)	1 (9.1%)			
	Combined	7 (35%)	9 (45%)	4 (20%)			
		Video files	Audio Files	PDFs	None		
Which resources did you use within the modules? Select all that apply.	Cohort 1	6 (54.6%)	0 (0%)	5 (45.5%)	0 (0%)		
	Cohort 2	9 (60%)	9 (60%)	13 (86.7%)	1 (6.7%)		
	Combined	15 (57.7%)	9 (34.6%)	18 (69.2%)	1 (3.9%)		
		Extremely difficult	Somewhat difficult	Neither	Somewhat easy	Extremely easy	
How did you find accessing the files through the links provided?	Cohort 1	0 (0%)	1 (9.1%)	2 (18.2%)	1 (9.1%)	7 (63.6%)	
	Cohort 2	0 (0%)	2 (13.3%)	3 (20%)	4 (26.7%)	6 (40%)	
	Combined	0 (0%)	3 (11.5%)	5 (19.2%)	5 (19.2%)	13 (50%)	
		Not at all	Slightly	Moderately	Very	Extremely	
How effective did you find the mindfulness and meditation practices included in the curriculum?	Cohort 1	0 (0%)	0 (0%)	2 (18.2%)	8 (72.7%)	1 (9.1%)	
	Cohort 2	0 (0%)	0 (0%)	3 (20%)	5 (33.3%)	7 (46.7%)	
	Combined	0 (0%)	0 (0%)	5 (19.2%)	13 (50%)	8 (30.8%)	
		Extremely dissatisfied	Somewhat dissatisfied	Neither	Somewhat satisfied	Extremely satisfied	
How would you rate your overall experience with the curriculum?	Cohort 1	0 (0%)	0 (0%)	0 (0%)	4 (36.3%)	7 (63.6%)	
	Cohort 2	0 (0%)	0 (0%)	2 (13.3%)	3 (20%)	10 (66.7%)	
	Combined	0 (0%)	0 (0%)	2 (7.7%)	7 (26.9%)	17 (65.4%)	
		Not at all	Slightly	Somewhat	Fairly	Completely	
I feel confident about implementing the practices I learned in this training in my daily work.	Cohort 1	0 (0%)	0 (0%)	1 (9.1%)	5 (45.5%)	5 (45.5%)	
	Cohort 2	0 (0%)	0 (0%)	2 (13.3%)	8 (53.3%)	5 (33.3%)	
	Combined	0 (0%)	0 (0%)	3 (11.5%)	13 (50%)	10 (38.6%)	
		Strongly disagree	Somewhat disagree	Neither	Somewhat agree	Strongly agree	
I would recommend this training to my colleagues.	Cohort 1	0 (0%)	0 (0%)	0 (0%)	3 (27.3%)	8 (72.7%)	
	Cohort 2	0 (0%)	0 (0%)	0 (0%)	4 (26.7%)	11 (73.3%)	
	Combined	0 (0%)	0 (0%)	0 (0%)	7 (26.9%)	19 (73.1%)	

Some participants skipped or left certain variables blank, resulting in fewer responses. Percentages are calculated out of total responses to each question.

## Data Availability

The datasets used and/or analyzed during the current study include sensitive and confidential information; therefore, the dataset cannot be shared.

## Supplementary Materials

The following supporting information can be downloaded at: https://www.mdpi.com/article/10.3390/healthcare14172735/s1, Supplementary Material S1: Mini-Z Worklife and Burnout Reduction Instrument; Supplementary Material S2: Knowledge, Skills, and Attitudes Survey; Supplementary Material S3: Engagement Survey; Supplementary Material S4: Supplementary Table with Cohen’s h Effect Size.

## Abbreviations

The following abbreviations are used in this manuscript:

CA AHEC	California Area Health Education Centers
HCW	Healthcare Worker
IPW	Institute for Professional Worklife
TtT	Train-the-Trainer
TCIM	Traditional Complementary and Integrative Medicine
KSA	Knowledge, Skills, and Attitudes
LOCF	Last Observation Carried Forward

## References

[1] Burn-Out an “Occupational Phenomenon”: International Classification of Diseases. https://www.who.int/news/item/28-05-2019-burn-out-an-occupational-phenomenon-international-classification-of-diseases.

[2] Edú-Valsania S., Laguía A., Moriano J.A. (2022). Burnout: A Review of Theory and Measurement. Int. J. Environ. Res. Public Health.

[3] Office of the Surgeon General (OSG) (2022). Addressing Health Worker Burnout: The U.S. Surgeon General’s Advisory on Building a Thriving Health Workforce.

[4] Linzer M., Jin J.O., Shah P., Stillman M., Brown R., Poplau S., Nankivil N., Cappelucci K., Sinsky C.A. (2022). Trends in Clinician Burnout with Associated Mitigating and Aggravating Factors During the COVID-19 Pandemic. JAMA Health Forum.

[5] Longo B.A., Schmaltz S.P., Williams S.C., Shanafelt T.D., Sinsky C.A., Baker D.W. (2023). Clinician Well-Being Assessment and Interventions in Joint Commission–Accredited Hospitals and Federally Qualified Health Centers. Jt. Comm. J. Qual. Patient Saf..

[6] Cauley A.W., Green A.R., Gardiner P.M. (2023). Lessons Learned from Clinicians in a Federally Qualified Health Center: Steps Toward Eliminating Burnout. J. Integr. Complement Med..

[7] Franco P.L., Christie L.M. (2021). Effectiveness of a One Day Self-Compassion Training for Pediatric Nurses’ Resilience. J. Pediatr. Nurs..

[8] Tarantino B., Earley M., Audia D., D’Adamo C., Berman B. (2013). Qualitative and Quantitative Evaluation of a Pilot Integrative Coping and Resiliency Program for Healthcare Professionals. Explore.

[9] Johnson J.R., Emmons H.C., Rivard R.L., Griffin K.H., Dusek J.A. (2015). Resilience Training: A Pilot Study of a Mindfulness-Based Program with Depressed Healthcare Professionals. Explore.

[10] Klockner K., Crawford C., Craigie M., Tsai L., Hegney D. (2021). A qualitative exploration of a mindful resiliency program for community healthcare providers. Nurs. Health Sci..

[11] Sood A., Sharma V., Schroeder D.R., Gorman B. (2014). Stress Management and Resiliency Training (SMART) program among Department of Radiology faculty: A pilot randomized clinical trial. Explore.

[12] Scheuch I., Peters N., Lohner M.S., Muss C., Aprea C., Fürstenau B. (2021). Resilience Training Programs in Organizational Contexts: A Scoping Review. Front. Psychol..

[13] Fowkes V., Blossom H.J., Mitchell B., Herrera-Mata L. (2014). Forging Successful Academic-Community Partnerships with Community Health Centers: The California Statewide AHEC Experience. Acad. Med..

[14] The Andrew Weil Center for Integrative Medicine What Is Integrative Medicine?. https://integrativemedicine.arizona.edu/about/definition.html.

[15] Scheid A., Dyer N.L., Dusek J.A., Khalsa S.B.S. (2020). A Yoga-Based Program Decreases Physician Burnout in Neonatologists and Obstetricians at an Academic Medical Center. Workplace Health Saf..

[16] Duarte J., Pinto-Gouveia J. (2016). Effectiveness of a mindfulness-based intervention on oncology nurses’ burnout and compassion fatigue symptoms: A non-randomized study. Int. J. Nurs. Stud..

[17] Cocchiara R.A., Peruzzo M., Mannocci A., Ottolenghi L., Villari P., Polimeni A., Guerra F., La Torre G. (2019). The Use of Yoga to Manage Stress and Burnout in Healthcare Workers: A Systematic Review. J. Clin. Med..

[18] La Torre G., Raffone A., Peruzzo M., Calabrese L., Cocchiara R.A., D’egidio V., Leggieri P.F., Dorelli B., Zaffina S., Mannocci A. (2020). Yoga and Mindfulness as a Tool for Influencing Affectivity, Anxiety, Mental Health, and Stress among Healthcare Workers: Results of a Single-Arm Clinical Trial. J. Clin. Med..

[19] Janssen M., Heerkens Y., Kuijer W., van der Heijden B., Engels J. (2018). Effects of Mindfulness-Based Stress Reduction on employees’ mental health: A systematic review. PLoS ONE.

[20] Sprang G., Gusler S., LaJoie S., Eslinger J., Smith E. (2023). Using Project ECHO to Keep Professionals Well at Work: Individual and Organizational Outcomes. Acad. Psychiatry.

[21] Linzer M., McLoughlin C., Poplau S., Goelz E., Brown R., Sinsky C. (2022). for the AMA-Hennepin Health System (HHS) burnout reduction writing team. The Mini Z Worklife and Burnout Reduction Instrument: Psychometrics and Clinical Implications. J. Gen. Intern. Med..

[22] Derrick B., Russ B., Toher D., White P. (2017). Test statistics for the comparison of means for two samples that include both paired and independent observations. J. Mod. Appl. Stat. Methods.

[23] Brown R. (2026). A Wald-Type Test for Partially Paired Binary Proportions: Extending the Derrick et al. Framework to Correlated Proportions.

[24] Naeem M., Ozuem W., Howell K., Ranfagni S. (2023). A Step-by-Step Process of Thematic Analysis to Develop a Conceptual Model in Qualitative Research. Int. J. Qual. Methods.

[25] Braun V., Clarke V. (2006). Using thematic analysis in psychology. Qual. Res. Psychol..

[26] Park E.Y. (2021). Meta-Analysis of Factors Associated with Occupational Therapist Burnout. Occup. Ther. Int..

[27] Luberto C.M., Wasson R.S., Kraemer K.M., Sears R.W., Hueber C., Cotton S. (2017). Feasibility, Acceptability, and Preliminary Effectiveness of a 4-Week Mindfulness-Based Cognitive Therapy Protocol for Hospital Employees. Mindfulness.

[28] Rees C.S., Craigie M.A., Slatyer S., Crawford C., Bishop M., McPhee E., Hegney D.G. (2020). Pilot study of the effectiveness of a Mindful Self-Care and Resiliency program for rural doctors in Australia. Aust. J. Rural Health.

[29] Lamothe M., Rondeau É., Malboeuf-Hurtubise C., Duval M., Sultan S. (2016). Outcomes of MBSR or MBSR-based interventions in health care providers: A systematic review with a focus on empathy and emotional competencies. Complement. Ther. Med..

[30] Rees C., Craigie M., Slatyer S., Heritage B., Harvey C., Brough P., Hegney D. (2018). Mindful Self-Care and Resiliency (MSCR): Protocol for a pilot trial of a brief mindfulness intervention to promote occupational resilience in rural general practitioners. BMJ Open.

[31] Stemley M. (2022). Reignite: Fighting Burnout with a Virtual Resiliency Program for Nurse Leaders. Nurse Lead..

[32] Yi-Frazier J.P., O’Donnell M.B., Adhikari E.A., Zhou C., Bradford M.C., Garcia-Perez S., Shipman K.J., Hurtado S.E., Junkins C.C., O’Daffer A. (2022). Assessment of Resilience Training for Hospital Employees in the Era of COVID-19. JAMA Netw. Open..

[33] Brennan J., McGrady A., Tripi J., Sahai A., Frame M., Stolting A., Riese A. (2019). Effects of a resiliency program on burnout and resiliency in family medicine residents. Int. J. Psychiatry Med..

[34] Mugford H., O’Connor C., Danelson K., Popoli D. (2022). Medical Students’ Perceptions and Retention of Skills from Active Resilience Training. Fam. Med..

[35] Zhang M., Murphy B., Cabanilla A., Yidi C. (2021). Physical relaxation for occupational stress in healthcare workers: A systematic review and network meta-analysis of randomized controlled trials. J. Occup. Health.

[36] Ungar M. (2011). The social ecology of resilience: Addressing contextual and cultural ambiguity of a nascent construct. Am. J. Orthopsychiatry.

[37] Ranjbar N., Erb M., Mohammad O., Moreno F.A. (2020). Trauma-Informed Care and Cultural Humility in the Mental Health Care of People from Minoritized Communities. Focus J. Lifelong Learn Psychiatry.

[38] Henritze E., Goldman S., Simon S., Brown A.D. (2023). Moral injury as an inclusive mental health framework for addressing climate change distress and promoting justice-oriented care. Lancet Planet Health.

[39] Sakallaris B.R., MacAllister L., Voss M., Smith K., Jonas W.B. (2015). Optimal Healing Environments. Glob. Adv. Health Med..

[40] Itchhaporia D. (2021). The Evolution of the Quintuple Aim. J. Am. Coll. Cardiol..

[41] Linzer M., Poplau S., Grossman E., Varkey A., Yale S., Williams E., Hicks L., Brown R.L., Wallock J., Kohnhorst D. (2015). A Cluster Randomized Trial of Interventions to Improve Work Conditions and Clinician Burnout in Primary Care: Results from the Healthy Work Place (HWP) Study. J. Gen. Intern. Med..

[42] Martín M., Ambriz L., Gonzalez Ramirez M., Borchers R., Schulson L.B., Cervantes L., Fernández A. (2026). Moral Injury Among Physicians Caring for Immigrant Patients Amid Anti-Immigrant Policies. JAMA Intern. Med..

[43] Horstman C., Lewis C., Bryan A., Federman S. Community Health Centers’ Progress and Challenges in Meeting Patients’ Essential Primary Care Needs: Findings from the Commonwealth Fund 2024 National Survey of Federally Qualified Health Centers. The Commonweath Fund. https://www.commonwealthfund.org/publications/issue-briefs/2024/aug/community-health-centers-meeting-primary-care-needs-2024-FQHC-survey.

[44] Fernandez H., Ayo-Vaughan M., Zephyrin L.C., Block R. (2024). Revealing Disparities: Health Care Workers’ Observations of Discrimination Against Patients. The Commonweath Fund. https://www.commonwealthfund.org/publications/issue-briefs/2024/feb/revealing-disparities-health-care-workers-observations.

[45] Usset T.J., Baker L.D., Griffin B.J., Harris J.I., Shearer R.D., Munson J., Godzik C., Torrey W.C., Bardach S.H., Mulley A.G. (2024). Burnout and turnover risks for healthcare workers in the United States: Downstream effects from moral injury exposure. Sci. Rep..

[46] American Psychological Association (2024). Stress in America 2024: A Nation in Political Turmoil. https://www.apa.org/pubs/reports/stress-in-america/2024/index.

