# Supplementary material for: Healing from the Heart: A Burnout Prevention and Resilience Curriculum Innovation for Health Professionals in Underserved Areas
Source: Healthcare (Basel). 2026 Aug 27;14(17):2735. doi: 10.3390/healthcare14172735 (PMC13565039; doi:10.3390/healthcare14172735)
Supplement: Supplementary file 1 [file healthcare-14-02735-s001.zip › Supplementary Material S1 Mini-Z Worklife and Burnout Reduction Instrument.pdf]

Z Zero Burnout Program

Mini Z base questionnaire for staff and trainees.

For questions 1-10, please indicate the best answer. [Note: do not include scoring in administration of survey]

1. Overall, I am satisfied with my current worklife: [Scoring: Responses 1-2 = satisfied]

1-Agree strongly 2-Agree 3-Neither agree nor disagree 4-Disagree 5-Strongly disagree

2. I feel a great deal of stress because of my work [Scoring: Responses 1-2 = high stress]

1-Agree strongly 2-Agree 3-Neither agree nor disagree 4-Disagree 5-Strongly disagree

****3. Using your own definition of "burnout", please select one of the answers below:** [Scoring: responses 3-5 = burnout]

1. I enjoy my work. I have no symptoms of burnout.
2. I am under stress, and don't always have as much energy as I did, but I don't feel burned out.
3. I am definitely burning out and have one or more symptoms of burnout, e.g. emotional exhaustion.
4. The symptoms of burnout that I'm experiencing won't go away. I think about work frustrations a lot.
5. I feel completely burned out. I am at the point where I may need to seek help.

~~~~~~

Comment for respondents: If you selected 4 or 5 on the burnout question (burnout symptoms won't go away, or completely burned out), please contact your Employee Assistance Program (EAP), contact info… If needed, the National Suicide Lifeline is available at (988). Even with less burnout (burnout symptoms present, response #3), you should feel free to reach out for support.

~~~~~~

4. My control over my workload is: [Scoring: Responses 3-5 = satisfactory control]

1 – Poor 2 – Marginal 3 – Satisfactory 4 – Good 5 – Optimal

5. My ability to complete work during the workday is: [Scoring: Responses 3-5 = satisfactory time to complete work]

1 – Poor 2 – Marginal 3 – Satisfactory 4 – Good 5 – Optimal

6. Which number best describes the atmosphere in your primary work area? [Scoring: Responses 4-5 = chaos]

1-Calm 2-Not too busy 3- Busy, but reasonable 4-Very busy 5- Hectic, chaotic

7. My professional values are well aligned with those of my leaders, medical director or program director: [Scoring: Responses 1-2 = high values alignment]

1-Agree strongly 2-Agree 3-Neither agree nor disagree 4-Disagree 5-Strongly disagree

8. The degree to which my care team works efficiently together is: [Scoring: Responses 3-5 = good teamwork]

1 – Poor 2 – Marginal 3 – Satisfactory 4 – Good 5 – Optimal

9. How often do you encounter negative experiences at work due to your <u>gender</u> (e.g. being denied work opportunities, being isolated or treated as if you were not competent, experiencing repeated, small slights at work, or other forms of discrimination)? [Scoring: Responses 1-2 = experiencing discrimination]

1-Frequently 2-Fairly often 3-Infrequently 4-Rarely 5-Never

10. How often do you encounter negative experiences at work due to your race (e.g. being denied work opportunities, being isolated or treated as if you were not competent, experiencing repeated, small slights at work, or other forms of discrimination)? [Scoring: Responses 1-2 = experiencing discrimination]

1-Frequently 2-Fairly often 3-Infrequently 4-Rarely 5-Never

Optional **additional questions for all respondents:**

11. What is the likelihood that you will leave your current job (at NAME) within TWO YEARS? [Scoring: Responses 3-5 = likely to leave work]

1-None 2-Slight 3-Moderate 4-Likely 5-Definitely

12. I feel valued by my organization/program/professional school:

Strongly disagree to strongly agree

Please tell us more about stresses and what may minimize them:

Additional questions based on role:

Additional questions for Professional Students and Residents/fellows:

How have the following items impacted your work-related satisfaction over the past month?
[Scoring: Add all the response numbers, range 5-25, >=20 is a positive and healthy resident experience]

1.Work interruptions (e.g. pages greater than expected, etc.)

1 – Al lot 2- A moderate amount 3- Somewhat 4- A little 5- Not at all

2. Lack of sleep

1 – Al lot 2- A moderate amount 3- Somewhat 4- A little 5- Not at all

3. Positive relationships with clinical support staff

1-Not at all 2-A little 3-Somewhat 4-A moderate amount 5- A lot (in a good way)

4. Support by peers

1-Not at all 2-A little 3-Somewhat 4-A moderate amount 5- A lot (in a good way)

5. Recognition by my school program

1-Not at all 2-A little 3-Somewhat 4-A moderate amount 5- A lot (in a good way)

Additional questions for administrative leaders (ad-mini)

1. I have the following amount of trust in my organization: [Scoring: Responses 3-5 = trust present]

1-None 2 – Minimal 3- Some 4-Moderate amount 5 – A great deal

2. I have the skills necessary to complete my administrative work. [Scoring: Responses 4-5 = skilled for role]

1-Strongly Disagree 2-Disagree 3-Neither Agree nor Disagree 4-Agree 5-Strongly Agree

3. I feel valued by my healthcare institution for the <u>administrative</u> work I do. [Scoring: Responses 4-5 = feel valued]

1-Strongly Disagree 2-Disagree 3-Neither Agree nor Disagree 4-Agree 5-Strongly Agree

4. I have adequate authority to make necessary decisions. [Scoring: Responses 4-5 = adequate authority]

1-Strongly Disagree 2-Disagree 3-Neither Agree nor Disagree 4-Agree 5-Strongly Agree

5. What is the likelihood of you choosing to leave your <u>administrative</u> role within two years? [Scoring: Responses 3-5 = likely to leave work]

1 –None 2-Slight 3-Moderate 4-Likely 5-Definitely

Demographic questions – may modify language as needed,

1. **Which of the following best describes you (choose one)? Please note, some roles have been grouped together to avoid small group sizes and protect anonymity.**

   Clinical

   a. Advanced Practice Provider
   b. Child and Family Services
   c. Fellow/ Resident – *If yes to this, receive additional questions (mini-Rez)*
   d. Professional student (specify_______)
   e. Imaging
   f. Medical Assistant/ Nursing Assistant/ Health Unit Coordinator
   g. Nurse
   h. Occupational, Physical or Speech Therapist, Audiologist
   i. Orthotics, Prosthetics, Seating, and Casting
   j. Pharmacy Services
   k. Physician
   l. Respiratory Care
   m. Other clinical – e.g. dental, neurodiagnostics, Motion Lab (fill in): _____

   Non-clinical

   n. Environment of Care
   o. Charge Integrity
   p. Information Systems
   q. Patient Access/Registration/Administrative Assistant
   r. Research department
   s. Supply Chain
   t. Other non-clinical – (fill in): _____

2. **Do you have an administrative leadership role at NAME? (e.g., AMD, supervisor, executive)**

   a. Yes – ***If yes to this, receive additional questions (Ad-Mini) and follow up question***
   b. No

3. (IF yes to #2) - What is your current leadership role?

a. Executive or senior leader
b. Associate Medical Director
c. Director or manager
d. Supervisor
e. Other (fill in)

4. Please tell us with which gender you identify, *optional* (select all that apply):
a. Male
b. Female
c. Non-binary
d. Genderqueer
e. Self-describe/not listed
f. Prefer not to answer

5. Please describe your race and ethnicity, *optional* (select all that apply):
a. White
b. Black or African American
c. LatinX
d. Asian
e. Native Hawaiian or Other Pacific Islander
f. Native American or Alaska Native
g. Middle Eastern or North African
h. Other (specify_____________)
i. Prefer not to answer

6. How many years have you been in practice, in professional school, in residency/fellowship or in the workforce?
a. 1-5 years
b. 6-10 years
c. 11-15 years
d. 16-20 years
e. More than 20 years
f. N/A
