# Supplementary material for: Healing from the Heart: A Burnout Prevention and Resilience Curriculum Innovation for Health Professionals in Underserved Areas
Source: Healthcare (Basel). 2026 Aug 27;14(17):2735. doi: 10.3390/healthcare14172735 (PMC13565039; doi:10.3390/healthcare14172735)
Supplement: Supplementary file 1 [file healthcare-14-02735-s001.zip › Supplementary Material S2 Knowledge, Skills, and Attitudes Survey.pdf]

AHEC Burnout Prevention Knowledge, Skills, & Attitudes Questionnaire – version 2022-23

	1 – Strongly Disagree	2 - Disagree	3 – Neither Agree/ Disagree	4 - Agree	5 – Strongly Agree
I can decrease the effect of workplace stressors on my personal and professional wellbeing.					
I feel well prepared to incorporate interventions that will help me retrieve the joy in my practice.					
I understand the importance of my well-being and how it directly affects my ability to care for others.					
I can recognize when I, or others around me, are exhibiting symptoms of burnout.					
I know the sources of burnout and feel empowered to address these issues in my workplace.					
I am confident in my ability to advocate for evidence-based changes to my work environment to promote provider and employee wellbeing.					

How do you rate **knowledge, comfort, and/or expertise** in the following topics:								
	**Before Course**				**After Course**			
	**None**<br>**1**	**Low**<br>**2**	**Moderate**<br>**3**	**High**<br>**4**	**None**<br>**1**	**Low**<br>**2**	**Moderate**<br>**3**	**High**<br>**4**
The mechanisms of burnout and prevention strategies.								
An understanding of why burnout prevention is crucial.								
An understanding in the relationship between preventing burnout in healthcare staff and patient outcomes.								
Recognizing signs and symptoms of burnout, as well as effective interventions to prevent it.								
Knowing definition of Moral Injury and how it leads to burnout.								
Identifying aspects from the Resilience Training that help you cope with stressors.								

1. How do you see preventing burnout in the workplace impacting the care you provide to patients?

2. Please rate the value of this session:

a. Not valuable
b. Somewhat valuable
c. Valuable
d. Extremely valuable

3. What was most helpful about this training?

4. How could the burnout prevention training be improved?
