# Supplementary material for: Healing from the Heart: A Burnout Prevention and Resilience Curriculum Innovation for Health Professionals in Underserved Areas
Source: Healthcare (Basel). 2026 Aug 27;14(17):2735. doi: 10.3390/healthcare14172735 (PMC13565039; doi:10.3390/healthcare14172735)
Supplement: Supplementary file 1 [file healthcare-14-02735-s001.zip › Supplementary Material S3 Engagement Survey.docx.pdf]

Resiliency Train the Trainer Post Engagement Survey

1. **Did you interact with any of the module files using the link that was sent to you? If so, select the modules you accessed through the links.**

○Module 1: Introduction to Resilience ○Module 2: The Mind ○Module 3: The Body ○Module 4: The Spirit ○Module 5: Environment and Community ○Module 6: System Change ○I interacted with all the modules through the link

2. **Which sessions did you find relevant to your work or personal development? Select all that apply.**

○Module 1: Introduction to Resilience ○Module 2: The Mind ○Module 3: The Body ○Module 4: The Spirit ○Module 5: Environment and Community ○Module 6: System Change ○I found them all relevant

3. **Were there modules that you found most exciting or impactful? Select all that apply.**

○Module 1: Introduction to Resilience ○Module 2: The Mind ○Module 3: The Body ○Module 4: The Spirit ○Module 5: Environment and Community ○Module 6: System Change

4. **Were you able to attend all the live sessions in January 2024?**

○Yes ○No

5. **Did you watch any of the recordings of the live sessions you missed?**

○Yes, all of them. ○Yes, some of them. ○No, I did not watch the recordings of the live sessions ○Other ____

6. **How do you feel about the date and time of the fall Zoom trainings? Is there a better time or date that works for you?**

Text entry.

7. **Which resources did you use within the modules? Select all that apply.**

○PDFs ○Audio files ○Video files

8. **How did you find accessing the files through the links provided?**

○Extremely difficult ○Somewhat difficult ○Neither easy nor difficult ○Somewhat easy ○Extremely easy

9. **How effective did you find the mindfulness and meditation practices included in the curriculum?**

○Not effective at all ○Slightly effective ○Moderately effective ○Very effective ○Extremely effective

10. **How effective did you find the mindfulness and meditation practices included in the curriculum?**

○Not effective at all ○Slightly effective ○Moderately effective ○Very effective ○Extremely effective

11. **How would you rate your overall experience with the curriculum?**

○Extremely dissatisfied ○Somewhat dissatisfied ○Neither satisfied nor dissatisfied ○Somewhat satisfied ○Extremely satisfied

12. **How did the training enhance your understanding of resilience and burnout prevention? Please explain.**

Text entry.

13. **Were there any practices or concepts that you have already started applying? If so, which ones?**

Text entry.

14. **Did you attend the in-person training?**

○Yes ○No

15. **How helpful was the in-person training component?**

○Not effective at all ○Slightly effective ○Moderately effective ○Very effective ○Extremely effective

16. **I feel confident about implementing the practices I learned in in this training in my daily work.**

○Not confident at all ○Slight confidence ○Somewhat confident ○Fairly confident ○Completely confident

17. **I would recommend this training to my colleagues.**

○Strongly disagree ○Somewhat disagree ○Neither agree nor disagree ○Somewhat agree ○Strongly agree

18. **I would recommend this training to my colleagues.**

○Strongly disagree ○Somewhat disagree ○Neither agree nor disagree ○Somewhat agree ○Strongly agree

19. **Are there any aspects of the training you found challenging or unclear?**

Text entry.

20. **What additional resources or topics would you like to see included in future trainings for this program?**

Text entry.

21. **Do you have any other comments or feedback you would like to share about your experience with the program?**

Text entry.
