# Supplementary material for: Healing from the Heart: A Burnout Prevention and Resilience Curriculum Innovation for Health Professionals in Underserved Areas
Source: Healthcare (Basel). 2026 Aug 27;14(17):2735. doi: 10.3390/healthcare14172735 (PMC13565039; doi:10.3390/healthcare14172735)
Supplement: Supplementary file 1 [file healthcare-14-02735-s001.zip › Supplementary Material S4 Supplementary Table with Cohen’s h Effect Size.pdf]

Supplemental Table: Cohen's *h* Effect size

Burnout Prevention Knowledge	Pre-Post1 (n1 = 37 vs n2 = 19)	Pre-Post2 (n1 = 37 vs n2 = 8)	Resilience Knowledge	Pre-Post1 (n1 = 37 vs n2 = 19)	Pre-Post2 (n1 = 37 vs n2 = 8)	Mini Z	Pre-Post1 (n1 = 37 vs n2 = 18)
I can decrease the effect of workplace stressors on my personal and professional wellbeing.	0.046	-0.309	I feel confident in my ability to bounce back, despite setbacks I may experience.	-0.150	-0.245	Overall, I am satisfied with my current job. (Agree, Strongly agree) SATISFACTION	0.021
I feel well prepared to incorporate interventions that will help me retrieve the joy in my practice.	-0.551	-0.165	I prioritize the importance of my personal well-being and relationship to myself.	0.032	-0.062	I feel a great deal of stress because of my job. (Agree, Strongly agree) STRESS	-0.349
I understand the importance of my well-being and how it directly affects my ability to care for others.	-0.012	-0.123	I am confident in my ability to successfully engage in mindfulness practices.	-0.701	-0.253	Using your own definition of "burnout", please select one of these answers. (Definitely, Won't go away, Completely) BURNOUT	-0.083
I can recognize when I, or others around me, are exhibiting symptoms of burnout.	-0.395	-0.489	I often check in with my own feelings throughout the day and offer myself compassion, even amid stressful days.	-0.739	-0.498	My control over my workload is: (Poor, Marginal) CONTROL	-0.062
I know the sources of burnout and feel empowered to address these issues in my workplace.	-0.132	-0.226	I am confident in my ability to implement healthy self-care rituals to support me, even in times of great stress.	-0.829	-0.767	My ability to complete work during the workday is: (Poor, Marginal) WORKLOAD	0.285
I am confident in my ability to advocate for evidence-based changes to my work environment to promote provider and employee wellbeing.	-0.239	-0.226	I am confident in my ability to advocate for evidence-based changes to my work environment to promote provider and employee wellbeing.	-0.513	-0.659	Which best describes the atmosphere in your primary work area? (Very busy, Hectic-chaotic) CHAOS	-0.115
The mechanisms of burnout and prevention strategies.	-1.926	-1.666	Understanding connections between mind, body, and environment which promote resilience.	-0.501	-0.309	My professional values are well aligned with those of my direct leaders. (Agree, Strongly agree) VALUES ALIGNED	-0.238
An understanding of why burnout prevention is crucial.	-1.212	-0.825	Realizing importance of sleep, nutrition, movement, and stress management for me and the care I give my patients.	-0.421	-0.278	The degree to which my team works efficiently together is (Poor, Marginal) TEAN WORK	-0.062
An understanding in the relationship between preventing	-1.779	-1.194	The definition of resilience and mindfulness interventions to promote resilience.	-0.806	-0.782	How often do you encounter negative experiences at work due	-0.172

burnout in healthcare staff and patient outcomes.						to your gender (e.g. being denied work opportunities)? (Frequently, Fairly Often) GENDER	
Recognizing signs and symptoms of burnout, as well as effective interventions to prevent it.	-1.311	-1.071	Recognizing symptoms of burnout and interventions to prevent it.	-0.701	-0.658	How often do you encounter negative experiences at work due to your race (e.g. being denied work opportunities)? (Frequently, Fairly Often) RACE	-0.186
Knowing definition of Moral Injury and how it leads to burnout.	-1.164	-0.671	Recognition of how neglecting our wellbeing may be harmful to our profession and patients.	-0.433	-0.046	I have the following amount of trust in my organization: TRUST	0.124
Can use resilience training to better cope	-2.101	-1.732	Knoows strategies to reduce trauma impact	-.618	-.388	I have the skills necessary to complete my administrative work. ADMIN SKILLS	-0.262
						I feel valued by my healthcare institution for the administrative work I do. FEEL VALUED	-0.228
						I have adequate authority to make necessary decisions. AUTHORITY	0.074
						What is the likelihood of you choosing to leave your administrative role within two years? LEAVE	-0.165
